# Assessment of affective dysregulation in children: development and evaluation of a semi-structured interview for parents and for children

**DOI:** 10.1186/s13034-024-00762-8

**Published:** 2024-06-20

**Authors:** Anne-Katrin Treier, Sara Zaplana Labarga, Claudia Ginsberg, Lea Teresa Kohl, Anja Görtz-Dorten, Ulrike Ravens-Sieberer, Anne Kaman, Tobias Banaschewski, Pascal-M. Aggensteiner, Charlotte Hanisch, Michael Kölch, Andrea Daunke, Veit Roessner, Gregor Kohls, Manfred Döpfner, the ADOPT consortium, Dorothee Bernheim, Stefanie Bienioschek, Maren Boecker, Daniel Brandeis, Kristina Butz, Jörg M. Fegert, Franziska Giller, Carolina Goldbeck, Martin Hellmich, Christine Igel, Michaela Junghänel, Anne Ritschel, Jennifer Schroth, Anne Schüller, Marion Steiner, Anne Uhlmann

**Affiliations:** 1 School of Child and Adolescent Cognitive Behavior Therapy (AKiP), Faculty of Medicine and University Hospital Cologne, University of Cologne, Cologne, Germany; 2 Department of Child and Adolescent Psychiatry, Psychosomatics and Psychotherapy, Faculty of Medicine and University Hospital Cologne, University of Cologne, Cologne, Germany; 3 Department of Child and Adolescent Psychiatry, Psychotherapy, and Psychosomatics, University Medical Center Hamburg-Eppendorf, Hamburg, Germany; 4 Department of Child and Adolescent Psychiatry and Psychotherapy, Central Institute of Mental Health, Medical Faculty Mannheim, University of Heidelberg, Mannheim, Germany; 5 Faculty of Human Sciences, University of Cologne, Cologne, Germany; 6 Department of Child and Adolescent Psychiatry/Psychotherapy, University Hospital Ulm, Ulm, Germany; 7 Department of Child and Adolescent Psychiatry, Neurology, Psychosomatics, and Psychotherapy, University Medical Center Rostock, Rostock, Germany; 8 Department of Child and Adolescent Psychiatry and Psychotherapy, TUD Dresden University of Technology, Dresden, Germany

**Keywords:** Affective dysregulation, Irritability, Children, Assessment, Clinical interview, Reliability, Validity

## Abstract

**Background:**

Children with affective dysregulation (AD) show an excessive reactivity to emotionally positive or negative stimuli, typically manifesting in chronic irritability, severe temper tantrums, and sudden mood swings. AD shows a large overlap with externalizing and internalizing disorders. Given its transdiagnostic nature, AD cannot be reliably and validly captured only by diagnostic categories such as disruptive mood dysregulation disorder (DMDD). Therefore, this study aimed to evaluate two semi-structured clinical interviews—one for parents and one for children.

**Methods:**

Both interviews were developed based on existing measures that capture particular aspects of AD. We analyzed internal consistencies and interrater agreement to evaluate their reliability. Furthermore, we analyzed factor loadings in an exploratory factor analysis, differences in interview scores between children with and without co-occurring internalizing and externalizing disorders, and associations with other measures of AD and of AD-related constructs. The evaluation was performed in a screened community sample of children aged 8–12 years (*n* = 445). Interrater reliability was additionally analyzed in an outpatient sample of children aged 8–12 years (*n* = 27).

**Results:**

Overall, internal consistency was acceptable to good. In both samples, we found moderate to excellent interrater reliability on a dimensional level. Interrater agreement for the dichotomous diagnosis DMDD was substantial to perfect. In the exploratory factor analysis, almost all factor loadings were acceptable. Children with a diagnosis of disruptive disorder, attention-deficit/hyperactivity disorder, or any disorder (disruptive disorder, attention-deficit/hyperactivity disorder, and depressive disorder) showed higher scores on the DADYS interviews than children without these disorders. The correlation analyses revealed the strongest associations with other measures of AD and measures of AD-specific functional impairment. Moreover, we found moderate to very large associations with internalizing and externalizing symptoms and moderate to large associations with emotion regulation strategies and health-related quality of life.

**Conclusions:**

The analyses of internal consistency and interrater agreement support the reliability of both clinical interviews. Furthermore, exploratory factor analysis, discriminant analyses, and correlation analyses support the interviews’ factorial, discriminant, concurrent, convergent, and divergent validity. The interviews might thus contribute to the reliable and valid identification of children with AD and the assessment of treatment responses.

**Trial registration:**

ADOPT Online: German Clinical Trials Register (DRKS) DRKS00014963. Registered 27 June 2018.

**Supplementary Information:**

The online version contains supplementary material available at 10.1186/s13034-024-00762-8.

## Introduction

Children with affective dysregulation (AD) typically show chronic irritability, severe temper tantrums, and sudden mood swings [1–3]. Emotion recognition and regulation develop from birth through interaction with a sensitive caregiver and lead to primary regulation strategies at the age of seven, which become more self-directed with increasing age [4]. Dysfunctions of emotion recognition and/or emotion regulation are suggested underlying mechanisms of AD, an assumption that is supported by findings of an elevated use of maladaptive emotion regulation strategies in children with AD [5]. In contrast to the concept of irritability—which solely comprises the proneness to anger [6]—AD additionally encompasses emotional reactivity such as anxiety, sadness, or positive emotions (e.g., exuberance; [3]).

However, there are various different operationalizations of AD. The fifth edition of the Diagnostic and Statistical Manual of Mental Disorders (DSM-5; [7]) introduced the diagnosis of disruptive mood dysregulation disorder (DMDD) as a categorical diagnosis for children with irritability and severe temper tantrums. In community samples, between 0.8% and 9% of all children and adolescents fulfill the diagnostic criteria for DMDD [8, 9], with lower rates in clinical ratings and higher rates in parent ratings. Furthermore, symptoms of AD in early childhood can be categorized under the disorder of dysregulated anger and aggression of early childhood based on the second revision of the Diagnostic Classification of Mental Health and Developmental Disorders of Infancy and Early Childhood (DC:0–5; [10]) from the age of 24 months onwards [11]. In the 11th revision of the International Classification of Diseases (ICD-11; [12]), AD is considered a diagnostic specifier of oppositional defiant disorder (ODD). According to the ICD-11, ODD can be defined with and without chronic irritability, due to the large overlap of diagnostic criteria between ODD and AD. Nevertheless, AD symptoms do not only occur in patients with ODD. On the contrary, children and adolescents with AD often show other externalizing disorders, especially attention-deficit/hyperactivity disorder (ADHD) but also conduct disorder (CD), as well as internalizing disorders, especially depressive and anxiety disorders [3, 8, 13].

Due to the overlap with this broad spectrum of other disorders, AD is also conceptualized as a transdiagnostic and dimensional rather than a distinct, categorical phenomenon [2, 14]. While the categorical classification of a disorder is useful in terms of guiding empirical research and decision-making such as treatment indication and selection, for the substantial number of patients with co-occurring disorders, such decisions might be more complicated [15]. In view of the large overlap of AD with both externalizing and internalizing disorders, a transdiagnostic and dimensional approach might therefore be more appropriate [14]. A stronger dimensional approach is also supported by the Research Domain Criteria (RDoC) initiative of the National Institute of Mental Health [16]. Within this initiative, AD fits well in the concept of frustrative non-reward in the negative emotionality domain [17].

In summary, we adopt a broader, dimensional, and transdiagnostic concept of AD, and perceive AD as excessive reactivity to emotionally positive or negative stimuli [2, 3]. Accordingly, AD comprises a proneness to a variety of emotional reactions, ranging from anger to anxiety or sadness, but also includes positive emotions such as exuberance.

Since the concept of AD is rather new, currently available instruments only assess particular aspects of AD: There are several questionnaires, which focus on irritability (Affective Reactivity Index, ARI; [18]), anger (Patient-Reported Outcome Measurement Information System Anger Scale, PROMIS; [19]), emotion regulation (Emotion Regulation Checklist, ERC; [20]), or DMDD (Diagnostic System for Mental Disorders in children and adolescents according to ICD-10 and DSM-5, DISYPS-III; [21]) Additionally, to assess the so-called dysregulation profile, there are two broadband questionnaires: the Child Behavior Checklist–Dysregulation Profile (CBCL-DP; [22]) and the Strengths and Difficulties Questionnaire–Dysregulation Profile (SDQ-DP; [23, 24]), with the dysregulation profile being defined as the co-existence of anxious/depressive, attention, and aggressive problems [22]. In both of these questionnaires, the profile is formed by combining specific subscales/items [22–24]. Finally, there are several clinical interviews, which focus on irritability (Clinician Affective Reactivity Index, CL-ARI; [25]) and DMDD (DMDD module of the Kiddie Schedule for Affective Disorders and Schizophrenia for School Aged Children Present and Lifetime Version, K-SADS-PL; [26], DMDD module of the Extended Strengths and Weaknesses Assessment of Normal Behavior, E-SWAN; [27]). Overall, however, comprehensive analyses of both the reliability and validity of instruments assessing AD are lacking (see the systematic review of measures assessing DMDD by [28]).

To date, the only comprehensive tool focusing on the broad conceptualization of AD is the German-language Diagnostic Tool for Affective Dysregulation in Children (DADYS; [3, 14, 29, 30]). The DADYS covers symptoms of irritability, impulsivity, temper outbursts, anger, mood swings, sadness, and exuberance. Besides a screening questionnaire (DADYS-Screen; [3, 30]), the DADYS comprises a parent, a teacher, and a child questionnaire (DADYS-PQ, DADYS-TQ, DADYS-CQ; [14, 29]), as well as semi-structured parent and child interviews (DADYS-PI, DADYS-CI; [29]). While the DADYS-Screen and the DADYS-PQ have already been comprehensively evaluated [3, 14, 30], the reliability and validity of the DADYS interviews have not yet been assessed.

Accordingly, the present study aimed to evaluate the DADYS interviews. The study was conducted in the context of the multicenter study ADOPT (Affective Dysregulation–Optimizing Prevention and Treatment), which integrates internationally established, highly experienced, and interdisciplinary research groups [2]. First, we analyzed the factor structure of the interviews by means of exploratory factor analyses. Second, we analyzed internal consistency and interrater reliability of the scales developed on the basis of factor analyses. Third, we aimed at demonstrating the validity of the DADYS interviews by analyzing their discriminative power to differentiate categorically between children with and without co-occurring internalizing and externalizing disorders (discriminant validity) as well as the dimensional associations with other measures of AD (concurrent validity) and with measures assessing emotion regulation strategies, externalizing and internalizing symptoms, and health-related quality of life (convergent and divergent validity).

## Methods

### Participants

For the evaluation of the DADYS interviews, two samples were used: A *screened community sample* (*n* = 445) was formed from a larger community sample (*n* = 9,759) which was recruited through residents’ registration offices in four German cities. Children aged 8 to 12 years were screened using the parent-rated DADYS-Screen [3, 30]. The age range of 8 to 12 years was chosen due to the ADOPT study’s focus on AD in childhood and because we wanted to administer the DADYS in both the clinical child interview and the child questionnaire. After screening for AD, children were classified as high AD (highest 10% raw scores; AD) or low AD (lowest 10% raw scores, NoAD). We chose this cut-off of 10% as an approximation of the prevalence rates of up to 9% reported in epidemiological studies [9]. The families of all children with AD and a random sample of the families of children without AD were invited to participate in a comprehensive assessment and were screened for additional inclusion criteria in one of the five study centers (child and adolescent psychiatric units or outpatient units). All families with AD fulfilling the additional criteria were invited to participate in the subsequent treatment study receiving either an AD-specific treatment or treatment as usual, while families without AD were subsequently monitored as a comparison group (ADOPT study, [2]). Additional inclusion/exclusion criteria were no participation in behavioral therapy focusing on AD, no autism spectrum disorder, and an intelligence quotient (IQ, based on clinical judgment) above 80. The assessment was completed online using the REDCap electronic data capture tool or offline in paper-and-pencil format. If parents provided permission, the DADYS interview was audio- or videotaped. For the assessment of interrater reliability, audio- or videotaped parent interviews (*n* = 246) were additionally rated by a blinded rater who was blind to the group status and the time of measurement.

An *outpatient sample* (*n* = 27) was recruited in the outpatient unit of the study center in Cologne for the analysis of interrater reliability of both the parent and the child interview in a clinical sample. All participating children had at least one diagnosis according to DSM-5. If parents and children provided permission, the DADYS interview was audio- or videotaped (*n* = 27). Participating families were at different stages of their psychotherapeutic treatment.

The unblinded interviewers at all study centers had a Bachelor’s or Master’s degree in psychology or education. All unblinded interviewers received standardized and extensive training on conducting the DADYS interviews, including practice videos for each item, sitting in on interviews, and conducting interviews under supervision. Blinded ratings and all ratings of the outpatient sample were conducted at both study sites in Cologne. Blinded raters for interrater agreement received the same extensive training, with the exception that they did not sit in on interviews or conduct interviews themselves. All unblinded interviewers and blinded raters were encouraged to consult their supervisor if they experienced any difficulties regarding the assessment with the DADYS interviews.

### DADYS parent and child interviews

The DADYS parent (DADYS-PI) and the parallel child interview (DADYS-CI) are semi-structured interviews which aim to calculate a dimensional score for AD. The DADYS-PI additionally allows for the categorical assessment of a DMDD diagnosis. To operationalize the broad conceptualization, we developed an item pool based on existing measures with different foci (ARI, [18], ERC, [20], DISYPS-III, [21], Conners’ Rating Scale, [31]). Items of the DISYPS-III and the ARI were combined due to item overlap. The original item pool comprised 35 items. For item selection, a Delphi rating of experts, and focus groups with experts and parents were implemented [30]. In the first step, items with very similar content, as rated by clinical experts in the field of AD (MD, AGD, URS), were deleted. In the second step, items were further reduced by conducting a focus group with clinical experts (clinicians and psychotherapists), a focus group with parents (outpatient clinic), and a focus group with children (outpatient clinic). The item set used in the sample for evaluating the interview consisted of 13 items assessing symptoms and five items assessing functional impairment for the DADYS-PI (see Table S1 in the supplement) as well as 10 items assessing symptoms and five items assessing functional impairment for the DADYS-CI.

In the two interviews, parents and children were asked to describe in detail the respective emotional or behavioral response defined in each item (e.g., exhibits strong mood swings, is able to delay gratification, is overly exuberant, is quick to anger) and to describe specific situations in which the response might be observed as well as the frequency of the response. Each item was rated by the clinician on a 4-point Likert scale ranging from 0 (age-appropriate/not present) to 3 (very strongly present). For each score, a brief description of the symptom severity was provided to aid clinical judgment. Items on functional impairment were only assessed if AD symptoms were present. For the assessment of the DMDD diagnosis in the DADYS-PI, the age of symptom onset, duration of symptoms, pervasiveness, and exclusion criteria were additionally assessed. Lastly, the severity of AD was rated globally with one item in the DADYS-PI.

### Measures for the validity assessment

**Affective dysregulation.** For the assessment of AD, two measures were used in addition to the DADYS interviews: (a) the DADYS parent questionnaire (DADYS-PQ) and child questionnaire (DADYS-CQ; [14, 29]) as well as (b) the Dysregulation Profile of the Child Behavior Checklist in its German version (CBCL-DP; [22, 32]). **DADYS-PQ/-CQ.** The DADYS questionnaires were also developed based on existing measures assessing aspects of AD (ARI, [18], PROMIS, [19], ERC, [20], DISYPS-III, [21], Conners’ Rating Scale, [31]), with an overlap with the original item pool of the DADYS interview. Items for the DADYS-Screen were selected and evaluated using a mixed-methods approach, including a Delphi rating of experts, focus groups with parents and experts, and psychometric analyses. Each item was rated on a 4-point Likert scale ranging from 0 (age-appropriate/not present) to 3 (very strongly present). For each questionnaire, the mean item score was calculated for the total symptoms scale (DADYS-PQ: 27 items; DADYS-CQ: 26 items) and for the functional impairment scale (DADYS-PQ: 5 items; DADYS-CQ: 5 items). In the current screened community sample, the internal consistency of each scale was sufficient to excellent (0.77 ≤ α ≤ 0.94). **CBCL-DP.** Parents rated the items of the subscales attention problems (10 items), aggressive behavior (18 items), and anxious/depressed (13 items) on a 3-point Likert scale ranging from 0 (not true) to 2 (very true or often true). We calculated the CBCL-DP scale by summing the mean item score for each subscale (range 0–2), which resulted in a range from 0 to 6 [33]. In the current screened community sample, the CBCL-DP scale showed excellent internal consistency (α = 0.94).

**Emotion regulation.** The Questionnaire for the Regulation of Frustration in Children was used to assess emotion regulation strategies (FRUST; [34, 35]). The questionnaire comprises the two subscales adaptive and maladaptive emotion regulation strategies, and is rated by parent (adaptive strategies: 10 items, maladaptive strategies: 4 items) and child (adaptive strategies: 33 items, maladaptive strategies: 7 items). The adaptive subscale includes strategies such as problem-solving or social support and the maladaptive subscale includes strategies such as rumination or avoidance. The items are rated on a 5-point Likert scale ranging from 0 (hardly ever) to 4 (almost always). We calculated a mean item score for each subscale. The internal consistency of the two subscales in the current screened community sample was sufficient to excellent (0.78 ≤ α ≤ 0.94).

**Externalizing/internalizing symptoms.** To assess the children’s internalizing and externalizing symptoms, we used the DISYPS-III [21]. Specifically, we employed the therapist-rated diagnostic screening checklists for internalizing symptoms (ILF-SCREEN-Internal, 19 items) and externalizing symptoms (ILF-SCREEN-External, 9 items), based on a parent interview. Additionally, we used the parent- and child-rated symptom checklists for ADHD (20 items) and disruptive disorders—including ODD, CD, DMDD, and callous-unemotional traits—(28 items). All items were rated on a 4-point Likert scale ranging from 0 (age-typical) to 3 (very strong). A mean item score across all items was calculated for each checklist, with the exception of the checklist for disruptive disorders, for which we calculated subscale scores for ODD (8 items), and CD (6 items). Since the items of the DMDD scale were part of the DADYS-PQ, we did not calculate this scale separately for the present study. In the current screened community sample, internal consistency was good to excellent for clinician-rated internalizing and externalizing symptoms as well as for parent- and child-rated ADHD and ODD symptoms (0.82 ≤ α ≤ 0.95), except for the parent- and child-rated CD scale (0.59 ≤ α ≤ 0.60) due to the diverse range of behaviors assessed in this scale.

Furthermore, due to the especially frequent co-occurrence of AD with both externalizing and affective disorders, we assessed diagnoses of ADHD, disruptive disorders (ODD and/or CD), and depressive disorder, coded as 0 (no) and 1 (yes), based on the DISYPS parent interviews. If the parents reported symptoms of these disorders on the screening checklist, we verified the respective diagnosis using the comprehensive checklist from the DISYPS-III.

**Quality of life. KIDSCREEN.** To assess health-related quality of life, we used the KIDSCREEN questionnaire [58], which measures subjective health and well-being in children and adolescents. Children completed the KIDSCREEN-10 Index (10 items) and parents completed the short version KIDSCREEN-27 (27 items). For both the child and parent version, items are rated on a 5-point Likert scale ranging from 1 (never/not at all) to 5 (always/very strong). The mean item score was calculated. Internal consistency in the current screened community sample was good to excellent (0.81 ≤ α ≤ 0.91).

### Statistical analyses

All statistical analyses were performed using SPSS Version 29 [36]. To reduce bias in the results, scales were only computed if at least 90% of the respective scale items were available [37].

**Sample characteristics.** Differences in sample characteristics between the AD and the NoAD subsample were examined using χ² tests for categorical variables, *t*-tests for interval-scaled variables, and Kruskal-Wallis tests for ordinal-scaled variables. As measures of effect size, we used Cramer’s V for χ² tests (0.10 ≤ ϕ_c_ < 0.30 small, 0.30 ≤ ϕ_c_ < 0.50 moderate, 0.50 ≤ ϕ_c_ large), Cohen`s *d* [38] for *t*-tests (0.20 ≤ *d* < 0.50 small, 0.50 ≤ *d* < 0.80 moderate, 0.80 ≤ *d* large), and Pearson correlations for Kruskal-Wallis tests (0.10 ≤ *r* <.30 small, 0.30 ≤ *r* <.50 moderate, 0.50 ≤ r large; [38]).

**Exploratory factor analysis.** For item reduction and scale development as well as the analysis of factor loadings (factorial validity), we performed an exploratory factor analysis for both the DADYS-PI and the DADYS-CI in the screened community sample. For each interview, we performed a principal component analysis (PCA) and a principal factor analysis (PFA). Only symptom items were included in the factor analysis since the functional impairment scale of the DISYPS-III was derived as a whole [21]. The Kaiser-Meyer-Olkin measure of sampling adequacy resulted in superb values (KMO_DADYS−PI_ = 0.94; KMO_DADYS−CI_ = 0.92). The scree test [39], the MAP test [40], and the parallel analysis [41] were used to determine the number of factors. Factor loadings of *a* ≥ 0.30 in the PCA and the PFA were considered robust [42].

**Scale characteristics.** To evaluate internal consistency, Cronbach’s alpha was calculated for the total symptom scale and the functional impairment scale for both interviews in the screened community sample, with values of *a* > 0.70 considered acceptable [43]. Furthermore, the corrected item-total correlation was calculated for each item, with values of *r* >.30 considered acceptable [44].

**Interrater reliability.** Interrater reliability for continuous AD symptoms was evaluated using intraclass correlations (ICC; [45]). Since the characteristics of the data differed between the screened community sample and the outpatient sample, we applied different models to calculate the ICC in the two samples. To compare unblinded interviewer ratings and blinded ratings in the screened community sample, we calculated the ICC one-way random-effects, absolute agreement model for single-rater ICC(1,1), as the multicenter design of the study did not allow for the same unblinded interviewers for all patients [46]. To compare the ratings in the outpatient sample, we calculated the ICC two-way random effects, absolute agreement, and single-rater ICC(2,1), as we used the same group of raters for all patients [46]. Single-rater models were applied in both samples as they more appropriately reflect routine clinical care, where one clinician usually conducts ratings [46]. For the interpretation of ICC values, we followed Koo and Li (ICC < 0.50 poor, 0.50 ≤ ICC ≤ 0.74 moderate, 0.75 ≤ ICC ≤ 0.89 good, ICC > 0.90 excellent; [46]). Interrater reliability for the dichotomous DMDD diagnosis based on the DADYS-PI was evaluated using Cohen’s kappa [47] in both samples. For the interpretation of ICC values, we followed Landis and Koch (κ < 0.20 slight, 0.21 ≤ κ ≤ 0.40 fair, 0.41 ≤ κ ≤ 0.60 moderate, 0.61 ≤ κ ≤ 0.80 substantial, κ > 0.80 almost perfect; [48]).

**Discriminant validity.** To analyze discriminant validity, we evaluated the differences between the DADYS interview scores of children with and without a diagnosis of disruptive disorders (ODD or CD), ADHD, depressive disorder, or an overarching diagnosis of any of these disorders using *t*-tests. Cohen`s *d* [38] was applied as a measure of effect size, using the interpretation mentioned above.

**Concurrent, convergent, and divergent Validity.** To analyze concurrent, convergent, and divergent validity, we calculated Pearson correlations between the DADYS interviews and comprehensive, parent- and child-rated measures of AD, parent- and child-rated measures of emotion regulation strategies, parent-, child-, and clinician-rated measures of externalizing and internalizing symptoms, and parent- and child-rated measures of health-related quality of life. Pearson correlation coefficients were interpreted as outlined above. Additionally, correlations accounting for at least 50% of the variance (*r* >.70) were classified as very large. Furthermore, we calculated paired *t*-tests for the comparison of mean differences between the two DADYS interviews and between the DADYS interviews and the DADYS questionnaire. Cohen`s *d* [38] was applied as a measure of effect size, with the aforementioned interpretation.

## Results

### Sample characteristics

The total screened community sample had a mean age of 10.70 years (*SD* = 1.32) and a mean socioeconomic status of 6.26 (*SD *= 1.20; range 1–7; value is based on the average national income obtained with the highest education and occupational qualification in the family; [49]) and 55.3% were boys. Approximately 32% of the children in the total screened community sample were diagnosed with ODD, CD, ADHD, or depressive disorder (see Table 1). When comparing the AD with the NoAD subsample of the screened community sample, we found a higher percentage of boys (small effect), lower age (small effect), and more co-occurring disorders (large effect) in the AD sample. The outpatient sample had a mean age of 10.41 years (*SD* = 1.45) and a mean socioeconomic status of 4.83 (*SD* = 1.76; range 1–7) and 81.5% were boys. Almost all children in the outpatient sample (90.9%) were diagnosed with ODD, CD, ADHD, or depressive disorder.

**Table 1:** Participant characteristics for the screened community sample including children with (AD) and without affective dysregulation (No-AD) and the outpatient sample

	Range	Screened community sample					Outpatient sample AD
		Total	AD	No-AD	Test statistic	Effect size	
		(*n* = 445)	(*n* = 265)	(*n* = 180)			(*n* = 27)
Gender (male): %		55.3%	62.6%	44.4%	χ²(1) = 14.36, *p* < .001	ϕ_c_ = 0.18	81.5%
Age (years): *M* (*SD*)	8–12	10.70 (1.32)	10.53 (1.28)	10.95 (1.34)	*t* (443) = 3.33, *p* = .001	*d* = 0.32	10.41 (1.45)
Socioeconomic status ^a^: *M* (*SD*)	1–7	6.26 (1.20)	6.20 (1.23)	6.35 (1.17)	*U* = 21266.50, *p* = .138	-	4.83 (1.76)
Diagnosis ODD, CD, ADHD, or DD: %		32.4%	54.3%	0%	χ²(1) = 144.61, *p* < .001	ϕ_c_ = 0.57	90.9%

Test statistics are based on χ² tests for categorical variables, *t*-tests for interval-scaled variables and Mann-Whitney U tests for ordinal-scaled variables. ϕ_c_ = effect size Cramer’s V for χ² tests. *d* = effect size Cohen’s *d* for *t*-tests. *M* = mean. *SD* = standard deviation. ODD = oppositional defiant disorder. CD = conduct disorder. ADHD = attention-deficit/hyperactivity disorder. DD = depressive disorder

^a^Value is based on the average national income obtained with the highest education and occupational qualification in the family [49]

### Exploratory factor analysis

The scree test [39], the MAP test [40], and the parallel analysis [41] pointed to a one-factor solution for both interviews. Therefore, we specified the number of factors to one factor, and all symptom items were combined in the total (AD) symptom scale. The AD factor explained 44.94% of the variance in the DADYS-PI and 47.53% of the variance in the DADYS-CI.

For the DADYS-PI, factor loadings ranged from 0.39 to 0.87 (*M* = 0.65, *SD* = 0.17) in the PCA and from 0.35 to 0.88 (*M* = 0.62, *SD* = 0.18) in the PFA (see Table 2). For the DADYS-CI, factor loadings ranged from 0.30 to 0.86 (*M* = 0.67, *SD* = 0.18) in the PCA and from 0.25 to 0.85 (*M* = 0.63, *SD* = 0.20) in the PFA. The lowest factor loadings in each analysis were found for the item “exuberance”. Since this item fell below our predefined robustness criterion of *a* = 0.30 [42] in only one of four analyses, and as we considered it important for the broader concept of AD, we decided to retain this item. All other items were considered robust and likewise retained.

**Table 2:** Item statistics, interrater reliability, factor loadings, item-total correlation for DADYS parent interview (DADYS-PI) and child interview (DADYS-CI)

Item	DADYS-PI							DADYS-CI					
	*M*^a^	*SD*^a^	ICC(1,1) ^b^	ICC(2,1)^c^	PCA^a^	PFA^a^	Item-total^a^	*M*^a^	*SD*^a^	ICC(2,1)^c^	PCA^a^	PFA^a^	Item-total^a^
**Mean total symptom scale**	0.83	0.61	0.94	0.94	-	-	-	0.69	0.53	0.87	-	-	-
01 cheerfulness (recoded)	0.74	0.76	0.66	0.76	0.69	0.64	0.61	0.72	0.68	0.57	0.61	0.54	0.51
02 mood swings	1.22	1.11	0.85	0.87	0.87	0.87	0.81	0.75	0.82	0.87	0.83	0.81	0.74
03 self-regulation (recoded)	1.01	0.97	0.70	0.46	0.62	0.57	0.54	1.00	0.90	0.71	0.62	0.55	0.55
04 delay of gratification (recoded)	0.92	1.04	0.69	0.58	0.58	0.54	0.51	0.97	1.04	0.49	0.45	0.39	0.38
05 demanding	1.00	1.03	0.72	-	0.70	0.66	0.63	-	-	-	-	-	-
06 clinginess	0.49	0.74	0.80	0.76	0.47	0.42	0.40	-	-	-	-	-	-
07 exuberance	0.33	0.67	0.84	0.86	0.39	0.35	0.34	0.21	0.49	0.76	0.30	0.25	0.24
08 verbalizing emotions (recoded)	0.87	0.96	0.79	0.71	0.43	0.38	0.36	-	-	-	-	-	-
09 sadness/ listlessness	0.37	0.66	0.82	0.84	0.50	0.46	0.43	0.49	0.65	0.54	0.63	0.58	0.52
10 temper tantrums	1.09	1.01	0.87	0.64	0.85	0.85	0.80	0.80	0.89	0.45	0.85	0.85	0.77
11 quick to anger	1.23	1.11	0.90	0.88	0.87	0.88	0.82	0.81	0.88	0.58	0.86	0.85	0.77
12 offended	0.87	0.97	0.85	0.94	0.75	0.72	0.68	0.55	0.73	0.83	0.76	0.73	0.66
13 irritability	0.64	0.87	0.86	0.91	0.74	0.71	0.66	0.57	0.74	0.95	0.77	0.73	0.67
**Mean functional impairment scale**	1.05	0.52	0.63	0.85	-	-	-	0.45	0.48	0.72	-		-
14 impaired relationships with family members	1.79	0.81	0.57	0.82	-	-	0.22	0.73	0.85	0.91	-		0.56
15 impaired relationships with adults	0.50	0.76	0.60	0.68	-	-	0.38	0.20	0.51	0.80	-		0.39
16 impaired relationships with children; limited leisure activities	0.82	0.96	0.76	0.85	-	-	0.40	0.32	0.65	0.39	-		0.47
17 impaired academic performance	0.68	0.87	0.62	0.62	-	-	0.33	0.19	0.50	0.66	-		0.45
18 strain	1.45	0.87	0.59	0.76	-	-	0.33	0.81	0.93	0.81	-		0.58

DADYS = Diagnostic Tool for Affective Dysregulation in Children. *M* = mean. *SD* = standard deviation. ICC(1,1) = intraclass correlation coefficient with one-way random effects model, absolute agreement, single measurement. ICC(2,1) = intraclass correlation coefficient with two-way random effects model, absolute agreement, single measurement. PCA = principal component analysis (loadings). PFA = principal factor analysis (loadings). item-total = corrected item-total correlation

^a^Screened community sample: DADYS-PI (total symptoms *n* = 445, functional impairment *n* = 262), DADYS-CI (total symptoms *n* = 444, functional impairment *n* = 376)

^b^Screened community sample: blind DADYS-PI (total symptoms *n* = 246, functional impairment *n* = 139)

^c^Outpatient sample: DADYS-PI (total symptoms *n* = 25, functional impairment *n* = 23), DADYS-CI (total symptoms *n* = 21, functional impairment *n* = 18)

### Scale characteristics

All items of the DADYS-PI and the DADYS-CI demonstrated the full scale range from 0 to 3. Item mean scores on the total symptom scale ranged from 0.33 (item “exuberance”) to 1.23 (item “quick to anger”) on the DADYS-PI and from 0.21 (item “exuberance”) to 1.00 (item “self-regulation”) on the DADYS-CI (see Table 2). Item mean scores on the functional impairment scale ranged from 0.45 (item “impaired relationships with adults”) to 1.79 (item “impaired relationships with family members”) on the DADYS-PI and from 0.19 (item “impaired academic performance”) to 0.81 (item “strain”) on the DADYS-CI.

Internal consistency for the total symptom scale was good in both interviews (DADYS-PI: α = 0.89; DADYS-CI: α = 0.87), whereas internal consistency for the functional impairment scale was sufficient in the DADYS-CI (α = 0.72) but insufficient in the DADYS-PI (α = 0.57). For the total symptom scale, item-total correlations were acceptable for all items in the DADYS-PI (0.34 ≤ *r* ≤ .82) and for all items in the DADYS-CI (0.38 ≤ *r* ≤ .77; see Table 2), with the exception of the item “exuberance” (*r* = .24). For the functional impairment scale, item-total correlations were acceptable for all items in the DADYS-PI (0.33 ≤ *r* ≤ .40), with the exception of the item “impaired relationships with family members” (*r* = .22), and for all items in the DADYS-CI (0.39 ≤ *r* ≤ .58).

### Interrater reliability

The total symptom scale demonstrated good to excellent interrater reliability (screened community sample: DADYS-PI ICC[1,1] = 0.94; outpatient sample: DADYS-PI ICC[2,1] = 0.94, DADYS-CI ICC[2,1] = 0.87; see Table 2). The functional impairment scale demonstrated moderate to good interrater reliability (screened community sample: DADYS-PI ICC[1,1] = 0.63; outpatient sample: DADYS-PI ICC[2,1] = 0.85, DADYS-CI ICC[2,1] = 0.72).

We found a substantial interrater agreement for the DMDD diagnosis based on the DADYS-PI in the screened community sample (κ = 0.73) and a perfect interrater agreement in the outpatient sample (κ = 1.00).

### Discriminant validity

Children with a disruptive disorder, ADHD, or any disorder (disruptive disorder, ADHD, or depressive disorder) scored higher than children without these disorders both on the DADYS-PI and the DADYS-CI (all large effects, see Table 3). As only 10 patients in the total sample showed a depressive disorder, we did not calculate the planned analyses for this disorder.

**Table 3:** Discriminant validity of DADYS parent interview (DADYS-PI) and child interview (DADYS-CI) in diagnostic subgroups

diagnosis		DADYS-PI ^a^					DADYS-CI ^a^				
		*n*	*M*	*SD*	Test statistic	*d*	*n*	*M*	*SD*	Test statistic	*d*
ODD/CD	No	336	0.63	0.54	*t*(304) = 19.03; *p* < .001	1.65	335	0.54	0.49	*t*(210) = 12.22; *p* < .001	1.25
	Yes	109	1.45	0.33			109	1.13	0.42		
ADHD	No	378	0.73	0.60	*t*(154) = 13.15; *p* < .001	1.19	377	0.61	0.52	*t*(105) = 8.21; *p* < .001	0.94
	Yes	67	1.40	0.33			67	1.09	0.42		
Any Diagnosis: ODD, CD, ADHD or DD	No	301	0.55	0.52	*t*(409) = 20.92; *p* < .001	1.82	336	0.63	0.54	*t*(304) = 19.03; *p* < .001	1.65
	Yes	144	1.40	0.33			109	1.45	0.33		

Test statistics are based on *t*-tests. *M* = mean. *SD* = standard deviation. *d* = Cohen’s d. DADYS = Diagnostic Tool for Affective Dysregulation in Children (Görtz-Dorten & Döpfner, 2021a). ODD = oppositional defiant disorder. CD = conduct disorder. ADHD = attention-deficit/hyperactivity disorder. DD = depressive disorder

^a^Total symptoms scale

### Concurrent, convergent, and divergent validity

The correlation between the DADYS-PI and the DADYS-CI was very large regarding the total symptom scales (*r* = .77) but moderate regarding the functional impairment scale (*r* = .31). Similarly, we found small mean differences between the DADYS-PI and the DADYS-CI for the total symptom scale (full DADYS-PI scale: *d* = 0.36; DADYS-PI cross-informant scale: *d* = 0.38) and moderate differences for the functional impairment scale (*d* = 0.72). Furthermore, the correlation between the total symptom scale and the functional impairment scale was moderate for the DADYS-PI (*r* = .42) and very large for the DADYS-CI (*r* = .74).

Associations between the DADYS interviews and other measures are presented in Table S2 in the Supplement. Regarding the association between the DADYS interviews (total symptom scale) and other measures of AD, we found large to very large correlations with parent- and child-rated DADYS questionnaires (total symptom scale) and with the parent-rated CBCL Dysregulation Profile (DADYS-PI: 0.64 ≤ *r* < .87; DADYS-CI: 0.64 ≤ *r* < .79; all *p* < .01). For the respective AD-specific functional impairment scale, we found small to large correlations between the DADYS interviews and the DADYS questionnaire (DADYS-PI: 0.26 ≤ *r* < .54; DADYS-CI: 0.49 ≤ *r* < .67; all *p* < .01). Furthermore, we found small to moderate mean differences between the DADYS interviews and the DADYS questionnaires for the total symptom scale (DADYS-PI/DADYS-PQ cross-informant scale: *d* = 0.48; DADYS-CI/DADYS-CQ cross-informant scale: *d* = 0.68), and small to no meaningful differences for the functional impairment scale (DADYS-PI/DADYS-PQ cross-informant scale: *d* = 0.19; DADYS-CI/DADYS-CQ cross-informant scale: *d* = 0.27).

Regarding the association between the DADYS interviews (total symptom scale) and parent- and child-rated emotion regulation strategies, the results revealed moderate to large positive correlations with maladaptive strategies (DADYS-PI: 0.41 ≤ *r* < .69; DADYS-CI: 56 ≤ *r* < .57; all *p* < .01) and moderate to large negative correlations with adaptive strategies (DADYS-PI: − 0.67 ≤ *r* < − .32; DADYS-CI: − 0.50 ≤ *r* < − .44; all *p* < .01).

Regarding the association between the DADYS interviews (total symptom scale) and externalizing symptoms, we found large to very large correlations with parent- and child-rated ODD symptoms (DADYS-PI: 0.58 ≤ *r* < .84; DADYS-CI: 0.67 ≤ *r* < .73; all *p* < .01), moderate to large correlations with parent- and child-rated CD symptoms (DADYS-PI: 0.41 ≤ *r* < .56; DADYS-CI: 0.42 ≤ *r* < .52; all *p* < .01), moderate to large correlations with parent- and child-rated ADHD symptoms (DADYS-PI: 0.47 ≤ *r* < .65; DADYS-CI: 0.53 ≤ *r* < .62; all *p* < .01), and large to very large correlations with clinician-rated externalizing symptoms (DADYS-PI: *r* = .78; DADYS-CI: *r* = .68; all *p* < .01). Regarding the association between the DADYS interviews (total symptom scale) and clinician-rated internalizing symptoms, correlations were moderate to large (DADYS-PI: *r* = .62; DADYS-CI: *r* = .48; all *p* < .01).

Finally, regarding parent- and child-rated health-related quality of life, we found moderate to large negative correlations with the total symptom scale of the DADYS interviews (DADYS-PI: − 0.65 ≤ *r* < −.43; DADYS-CI: − 0.58 ≤ *r* < − .53; all *p* < .01) and small to moderate negative correlations with the functional impairment scale of the DADYS interviews (DADYS-PI: − 0.35 ≤ *r* < − .20; DADYS-CI: − 0.46 ≤ *r* < − .43; all *p* < .01).

## Discussion

The aim of the present study was to evaluate the semi-structured clinical DADYS interviews for parents and children in a screened community sample of children with and without AD symptoms as well as in an outpatient sample. The results suggest that both the DADYS-PI and the DADYS-CI are promising and overall reliable and valid interviews for assessing AD in children.

For all items assessing symptoms of AD, we found one factor that provided the best fit to the data, both in the DADYS-PI and the DADYS-CI. Thus, all symptom items were combined into the total (AD) symptom scale. Additionally, the functional impairment scale of the DISYPS-III [21] was added to assess AD-specific functional impairment. As the DADYS-PI further allows for the assessment of a categorical DMDD diagnosis, the DADYS encompasses both the broader conceptualization of AD, implying the proneness to a variety of emotional reactions [2, 3], and the more specific DMDD diagnosis in accordance with the DSM-5 [7]. On a more methodological level, it also allows for both a dimensional assessment (AD symptoms, functional impairment) and a categorical assessment (DMDD) of AD. While a categorical approach might aid the decision-making process regarding the need for treatment, a dimensional approach brings several further advantages, such as a more comprehensive and individual assessment, a more precise assessment of treatment response, and less stigmatization [50, 51]. Finally, the DADYS allows not only for the assessment of AD in both a child and a parent interview, but also in parent, child, and teacher questionnaires.

Thus, the DADYS is able to assess AD both dimensionally and categorically, while other existing instruments are only able to assess AD either dimensionally (ARI, [18], PROMIS, [19], ERC, [20], CL-ARI, [25], E-SWAN, [27]) or categorically (K-SADS-PL, [26]). Only one other instrument besides the DADYS includes an AD-specific functional impairment scale – the CL-ARI [25]. Moreover, the DADYS is the only comprehensive tool focusing on the broad conceptualization of AD in parent, child, teacher, and clinical ratings, while the other measures offer either parent/child rating (ARI, [18], PROMIS, [19], ERC, [20], CBCL-DP, [22], SDQ-DP, [23, 24]) or clinician rating (CL-ARI,  [25], K-SADS-PL, [26], E-SWAN, [27]). Finally, comprehensive analyses of reliability and validity of other instruments assessing AD are still lacking [28]. Taken together, the DADYS offers a variety of advantages which have not yet been covered by any other instrument.

As indicators of reliability, we analyzed the internal consistency and the interrater reliability. Generally, we found acceptable to good internal consistencies for the DADYS interviews based on the samples analyzed, with the only exception being the functional impairment scale of the DADYS-PI. While Haller [25] reported an acceptable internal consistency for their irritability-related functional impairment scale (CL-ARI, α = 0.75), interestingly, Thöne, Gortz-Dorten [52] likewise found a lower internal consistency for the functional impairment scale used in the DADYS in a clinical interview assessing ADHD based on a parent interview (ILF-External, DISYPS-III, [21]). As an explanation for this finding, the latter authors argued that the items comprise rather heterogeneous aspects of functional impairment. Particularly in a nonclinical sample such as our screened community sample, if a child is angry, irritable, and moody within his/her own family, this might not be similarly the case in school, with peers, or with other adults. It should be noted that the impairment scale of the CL-ARI assesses functional impairment in three domains (i.e. family, school, and peers) while the impairment scale of the DADYS-PI contains, in addition to these three domains, functional impairment in contact with adults outside the family as well as the patient’s strain. Even though one item of the functional impairment scale of the DADYS-PI (“impaired relationships with family members”) showed an item-total correlation below *r* = .30, excluding this item did not improve the Cronbach’s alpha. Moreover, when interpreting the limited internal consistencies of the functional impairment scales, it should be considered that the sample for these scales consisted only of children with reported AD symptoms, which might have led to an underestimation of reliability and validity. When comparing the good internal consistencies of the DADYS total symptom scales (α = 0.87–89) with other clinical measures of DMDD symptoms (CL-ARI, α = 0.78–0.87; [25], K-SADS-PL, α = 0.92, [53]) and with parent-rated measures of AD (DADYS-PQ, α = 0.72–0.96, [14], DADYS-Screen, α = 0.94, [30]), our data showed mostly comparable internal consistencies.

Regarding interrater reliability, we applied different ICC models for the samples according to the characteristics of the respective data. In both samples, we found moderate to excellent interrater reliability for the total symptom scale and the functional impairment scale in the DADYS interviews. To the best of our knowledge, no study has examined the dimensional interrater reliability of AD symptoms in other clinical interviews. Thus, we were unable to compare the good to excellent interrater reliability of the DADYS total symptom scales (ICC[1,1] = 0.94, ICC[2,1] = 0.87–0.94) with other AD scales. When comparing the moderate to good interrater reliability of the DADYS functional impairment scales (ICC[1,1] = 0.63, ICC[2,1] = 0.72–0.85) with clinical measures of functional impairment in externalizing symptoms, we found lower scores than the ILF-External (DISYPS-III, ICC[1,1] = 0.89-0.92, [52]), which may be explained by the fact that the ILF-External was evaluated using a clinical sample. Interrater agreement for the dichotomous DMDD diagnoses was substantial to perfect (κ = 0.73 − 1.00). Compared to other measures of DMDD diagnoses, our values were comparable to slightly higher (K-SADS-PL, κ = 0.63, [54], Conners’ Rating Scale, κ = 0.68, [55]).

Taken together, our results on the internal consistency and interrater agreement of the DADYS-PI and the DADYS-CI for the scales (i.e. dimensional assessment) and for the DMDD diagnosis (i.e. categorical assessment) further strengthen our argument that the DADYS can reliably assess AD from both a dimensional and categorical perspective.

As indicators of validity, we analyzed factorial, discriminant, concurrent, convergent, and divergent validity. Regarding factorial validity, almost half of the variance was explained by the total symptom factor. All factor loadings were acceptable (0.38 to 0.88), except for the item exuberance (0.25–0.39). Comparing the factor loadings of the total symptom scales of the DADYS interviews with clinical measures of DMDD (K-SADS-PL, 0.52 to 0.90, [53]) and parent-rated measures of AD (DADYS-Screen, 0.64-0.86, [30], DADYS-PQ, 0.30–1.02, [35]), our data showed mostly comparable factor loadings. In order to capture the broader concept of AD and to be consistent with the DADYS parent questionnaire, we decided to retain the item exuberance.

The present findings further support the concurrent validity of the DADYS interviews: The DADYS interview total symptom scale showed the strongest associations with parent- and child-rated questionnaires of AD with similar item contents, and the DADYS interview functional impairment scale showed the strongest associations with parent- and child-rated measures of AD-specific functional impairment with the same item content. The associations were particularly strong when using the same informant source – that is the parent interview with the parent questionnaires (AD symptoms: *r* = .78–0.87, functional impairment with DADYS-PQ: *r* = .54) and the child interview with the child questionnaires (AD symptoms: *r* = .79, functional impairment: *r* = .67). The effects are comparable to larger than the associations found between the CL-ARI [25] and clinician-, youth-, and parent-rated measures of irritability (*r* = .42–0.89) and are comparable to the associations between the parent-rated DADYS-Screen [3] and parent- and child-rated measures of AD symptoms (*r* = .67–0.83).

Our analysis of associations with measures of externalizing and internalizing symptoms supports the discriminant, convergent, and divergent validity of the DADYS interviews. First, we found strong discriminative effects of the DADYS interviews for disruptive disorder, ADHD, and any disorder (disruptive, ADHD, and depressive disorder), which were comparable to or larger than the moderate to large discriminative effects found for the DADYS-Screen [3]. Second, we found moderate to very large associations of AD symptoms in the DADYS interview with internalizing and externalizing symptoms. These effects were comparable to or larger than the non-significant to large correlation coefficients reported for internalizing symptoms in the CL-ARI [25] and the moderate to very large correlation coefficients found for internalizing and externalizing symptoms in the DADYS-Screen [4]. When comparing the coefficients within each rater, both DADYS interviews showed the strongest correlations with ODD symptoms, followed by ADHD symptoms, and lastly CD symptoms in all parent-rated measures (*z* = 2.75–12.31, *p* = .000–0.006) and in most child-rated measures (*z* = 1.48–7.46, *p* = .000–0.139). Furthermore, we found a stronger correlation of externalizing symptoms compared to internalizing symptoms in clinical measures (*z* = 5.19–5.26, *p* < .001). These differential relationships are in line with previous research on associations of internalizing and externalizing symptoms both with DMDD symptoms [8, 13] and with AD symptoms (DADYS-Screen, [3]). The associations with both internalizing and externalizing symptoms further emphasize the necessity of the transdiagnostic conceptualization of AD.

Finally, AD symptoms in the DADYS interviews showed small to large associations with emotion regulation strategies and health-related quality of life, which is in line with the associations found with the DADYS-Screen [3].

Besides the evaluation of both the DADYS-PI and the DADYS-CI, it is important to highlight the differences between the two interviews. While AD symptoms showed a very large correlation between the two DADYS interviews, the correlation for functional impairment was only moderate. Similarly, we found small mean differences for the total scale and moderate differences for the functional impairment scale. As mentioned above, for many associations with validation measures, we found stronger effects when using the same informant source (i.e., parents vs. children). Taken together, our findings suggest that even though there are associations between clinical, parent, and child ratings, there are also some discrepancies among them. Earlier research evaluated these discrepancies as bias or lack of validity, but they are nowadays interpreted as reflecting the unique perspectives of the different informants, with each providing specific and additional information [56]. Thus, an additional strength of both DADYS interviews lies in the possibility to include both parents and children as sources of information in clinical ratings. Furthermore, as a comprehensive assessment tool, the DADYS additionally allows for the inclusion of parent, children and teacher ratings using questionnaires.

Limitations of this study include the restricted age range of 8–12 years within the evaluation, which does not allow for generalizations to children outside of this age range. Furthermore, the screened community sample showed a rather high mean socioeconomic status, which may suggest a lower willingness of parents with low or medium socioeconomic status to participate in the study, potentially reducing the representativeness of the findings. However, the most important limitation of the present study seems to be that the sample did not comprise the full spectrum of AD, but rather consisted of children in the lowest and the highest percentile on the continuum. Interestingly, previous analyses of the psychometric quality of the parent-rated DADYS-Screen [3] have already investigated a potential increase in effects when examining these extreme groups and found only small deviations between correlations for the total sample and the extreme groups. Since a possible overestimation of the reliability and validity of the DADYS interviews cannot currently be ruled out, the presented analyses and results of the DADYS interviews should be interpreted as promising indications of the reliability and validity of the instrument, but replication in further studies with children and adolescents with different levels of AD are needed in order to ultimately establish reliability and validity. Additionally, it should be kept in mind that the internal consistency of the impairment scale of the DADYS-PI must be rated as insufficient and that both the correlation between the symptom scale and the impairment scale of the DADYS-PI and the correlation between the impairment scales of the parent and child interviews were only moderate. In particular, since psychometric studies of related instruments have also found indications of limited psychometric quality of impairment scales (e.g., [52]), caution is warranted when using the impairment scale of the DADYS-PI, and further research on the functional impairment assessed by parents is needed. For example, it might be worthwhile to investigate whether parental burden has a significant influence on the perceived and reported functional impairment of the child. Lastly, the analyses were based on a cross-sectional design, and it would also be interesting to gain insight into longitudinal associations of child AD with later internalizing and externalizing symptoms (predictive validity, see e.g., [57]). Strengths of this study include the elaborate process of developing the DADYS interviews, the ability of the DADYS to assess AD dimensionally and categorically using the child and the parent as informants, the large screened community sample including children both with and without AD, the additional inclusion of an outpatient sample for interrater reliability, and finally, the inclusion of diverse perspectives for validity analyses (child, parent, and clinical ratings).

## Conclusions

This study contributes to the assessment and understanding of children with AD. We evaluated two newly developed semi-structured clinical DADYS interviews—one for parents and one for children. As such, the study is the first to evaluate a clinical interview assessing the broader transdiagnostic conceptualization of AD. Generally, our analyses of internal consistencies and interrater agreement support the reliability of both DADYS interviews. Furthermore, exploratory factor analyses, discriminant analyses, and correlation analyses support the factorial, discriminant, concurrent, convergent, and divergent validity of both DADYS interviews. Since the two DADYS interviews allow for both categorical and dimensional assessment and the inclusion of parents and children as informants, the measure might contribute to the identification of children with AD and the assessment of treatment response.

### Electronic supplementary material

Below is the link to the electronic supplementary material.

Supplementary Material 1

## Data Availability

Data are available upon reasonable request after the publication of the main results of the ADOPT study.

## Abbreviations

AD: Affective dysregulation
ADHD: Attention-deficit/hyperactivity disorder
ARI: Affective Reactivity Index
CBCL-DP: Child Behavior Checklist–Dysregulation Profile
CD: Conduct disorder
CL-ARI: Clinician Affective Reactivity Index
DADYS: Diagnostic Tool for Affective Dysregulation in Children
DADYS-CI: DADYS clinical child interview
DADYS-CQ: DADYS child questionnaire
DADYS-PI: DADYS clinical parent interview
DADYS-PQ: DADYS parent questionnaire
DADYS-Screen: DADYS screening questionnaire
DADYS-TQ: DADYS teacher questionnaire
DISYPS-III: Diagnostic System for Mental Disorders in children and adolescents according to ICD-10 and DSM-5
DMDD: Disruptive mood dysregulation disorder
DSM-5: Diagnostic and Statistical Manual 5th edition
ERC: Emotion Regulation Checklist
E-SWAN: Extended Strengths and Weaknesses Assessment of Normal Behavior
FRUST: Questionnaire for the Regulation of Frustration in Children
ICC: Intraclass correlations
ICC(1,1): ICC one-way random-effects, absolute agreement model for single rater
ICC(2,1): ICC two-way random effects, absolute agreement model for single rater
ICD-11: International Classification of Diseases 11th edition
KMO: Kaiser-Meyer-Olkin
K-SADS-PL: Kiddie Schedule for Affective Disorders and Schizophrenia for School Aged Children Present and Lifetime Version
NoAD: Children in the lowest 10% raw scores on the DADYS-Screen
ODD: Oppositional defiant disorder
PCA: Principal component analysis
PFA: Principal factor analysis
PROMIS: Patient-Reported Outcome Measurement Information System
RDoC: Research Domain Criteria
SDQ-DP: Strengths and Difficulties Questionnaire–Dysregulation Profile
