# Supplementary material for: Assessment of affective dysregulation in children: development and evaluation of a semi-structured interview for parents and for children
Source: Child Adolesc Psychiatry Ment Health. 2024 Jun 20;18:75. doi: 10.1186/s13034-024-00762-8 (PMC11191270; doi:10.1186/s13034-024-00762-8)
Supplement: Supplementary file 1 — Supplementary Material 1 [file 13034_2024_762_MOESM1_ESM.docx]

**Supplement**

**Assessment of Affective Dysregulation in Children: Development and Evaluation of a Semi-Structured Interview for Parents and for Children**

Anne-Katrin Treier, Sara Zaplana Labarga, Claudia Ginsberg, Lea Teresa Kohl, Anja Görtz-Dorten, Ulrike Ravens-Sieberer, Anne Kaman, Tobias Banaschewski, Pascal-M. Aggensteiner, Charlotte Hanisch, Michael Kölch, Andrea Daunke, Veit Roessner, Gregor Kohls, Manfred Döpfner on behalf of the ADOPT Consortium

**Table S1**

*Items of the DADYS parent interview (German and translation to English)*

| **no.** | **Original German item** | **English translation** | **short form** | **origin** |
| --- | --- | --- | --- | --- |
| 01 | Ist ___ ein fröhliches, meist gut gelauntes, ausgeglichenes Kind? | Is ___ a happy, mostly cheerful, well-adjusted child? | cheerfulness | ERC |
| 02 | Zeigt ___ starke Stimmungsschwankungen, wechselt ___ schnell oder häufig zwischen guter und schlechter Stimmung? | Does ___ exhibit strong mood swings, does ___ move quickly or frequently from positive to negative moods? | mood swings | ERC |
| 03 | Kann __ sich schnell wieder fangen, wenn ___ sich ärgert oder traurig ist? | Can __ recover quickly from episodes of upset or distress? | self-regulation | ERC |
| 04 | Kann ___ gut abwarten, bis sie/er eine Belohnung oder etwas Schönes bekommt? | Is ___ able to delay gratification or wait for good things? | delay of gratification | ERC |
| 05 | Muss den Forderungen von ___ sofort nachgekommen werden? | Do the demands of ___ need to be met immediately? | demanding | Conners 3 |
| 06 | Ist ___ weinerlich oder bei Eltern oder anderen Bezugspersonen anklammernd? | Is ___ whiny or clingy with parents or other caregivers? | clinginess | ERC |
| 07 | Zeigt __ positive Gefühle sehr ausgeprägt, sodass es andere stört? | Is __ overly exuberant in a way that disturbs others? | exuberance | ERC |
| 08 | Kann ___ eigene Gefühle benennen bzw. umschreiben? | Can ___ name or paraphrase their own feelings? | verbalizing emotions | ERC |
| 09 | Wirkt ___ traurig oder lustlos? | Does ___ seem sad or listless? | sadness / listlessness | ERC |
| 10 | Hat ___ schwere oder andauernde Wutausbrüche, die sich verbal (z.B. Schimpfwörter benutzen) oder im Verhalten (z.B. körperliche Aggressionen gegenüber Personen oder Gegenständen) zeigen? | Does ___ have severe or persistent temper outbursts that are expressed verbally (e.g., using swear words) or in behavior (e.g., physical aggression toward people or objects)? | temper tantrums | DISYPS/ARI |
| 11 | Wird ___ schnell wütend? | Is ___ quick to anger? | quick to anger | DISYPS/ARI |
| 12 | Ist ___ häufig beleidigt oder verärgert? | Is ___ often offended or annoyed? | offended | DISYPS/ARI |
| 13 | Ist ___ in seiner/ihrer Grundstimmung die meiste Zeit über gereizt? | Is ___ irritable in their basic mood most of the time? | irritability | DISYPS/ARI |
| 14 | Haben Sie den Eindruck, dass durch seine/ihre Reizbarkeit, Stimmungs-schwankungen, Wut die Beziehung von ___ zu Ihnen, seinen/ihren Geschwistern oder anderen Familienmitgliedern belastet oder beeinträchtigt wird? | Do you feel that their irritability, mood swings, anger is straining or affecting ___'s relationship with you, their siblings, or other family members? | impaired relationships with family members | DISYPS |
| 15 | Haben Sie den Eindruck, dass durch seine/ihre Reizbarkeit, Stimmungs-schwankungen, Wut die Beziehung von ___ zu Erwachsenen außerhalb der Familie (z. B. Lehrer/innen, Erzieher/innen, Trainer/innen) belastet oder beeinträchtigt wird? | Do you feel that because of their irritability, mood swings, anger, ___'s relationships with adults outside the family (e.g., [preschool] teachers, coaches) are strained or impaired? | impaired relationships with adults | DISYPS |
| 16 | Haben Sie den Eindruck, dass durch seine/ihre Reizbarkeit, Stimmungs-schwankungen, Wut die Beziehung von ___ zu anderen Kindern/Jugendlichen o-der die Beteiligung an Freizeitaktivitäten (z. B. Sport in Vereinen) belastet oder beeinträchtigt wird? | Do you feel that their irritability, mood swings, anger are straining or interfering with ___'s relationships with other children / adolescents or participation in leisure activities (e.g., sports in clubs)? | impaired relationships with children; limited leisure activities | DISYPS |
| 17 | Haben Sie den Eindruck, dass durch seine/ihre Reizbarkeit, Stimmungs-schwankungen, Wut die schulischen Leistungen von ___ belastet oder beeinträchtigt werden? | Do you have the impression that ___'s school performance is burdened or impaired by their irritability, mood swings, anger? | impaired academic performance | DISYPS |
| 18 | Haben Sie den Eindruck, dass ___ unter der Reizbarkeit, den Stimmungs-schwankungen und der Wut erheblich leidet? | Do you feel that ___ suffers significantly from their irritability, mood swings, and anger? | strain | DISYPS |

*Note.* DADYS = Diagnostic Tool for Affective Dysregulation in Children (Görtz-Dorten & Döpfner, 2021a); Conners 3 = Conners attention and behavior scales 3 (Lidzba et al., 2013); ERC = Emotion Regulation Checklist (Shields & Cicchetti, 1997); DISYPS = Diagnostic System for Mental Disorders in Children and Adolescents III; items translated (Döpfner & Görtz-Dorten, 2017). Items of the child interview were reworded to address the child directly. Items 6 and 8 were not assessed in the DADYS child interview.

**Table S2**

*Correlation analyses for DADYS parent (DADYS-PI) and child interview (DADYS-CI)*

| measure | rater | *DADYS-PI* | | *DADYS-CI* | |  |
| --- | --- | --- | --- | --- | --- | --- |
|  |  | total  symptoms | functional impairment | total  symptoms | functional impairment |  |
|  |  | *n* = 439-445 | *n* = 260-262 | *n* = 438-444 | *n* = 373-376 |  |
| correlations with other measures of affective dysregulation | | | | | | |
| DADYS-PQ: total symptoms | parent | .87** | .41** | .70** | .52** |  |
| DADYS-PQ: functional impairment | parent | .79** | .54** | .62** | .49** |  |
| DADYS-CQ: total symptoms | child | .64** | .11 | .79** | .65** |  |
| DADYS-CQ: functional impairment | child | .57** | .26** | .68** | .67** |  |
| CBCL: dysregulation profile | parent | .78** | .34** | .64** | .49** |  |
| correlations with measures of emotion regulation strategies^a^ | | | | | | |
| maladaptive strategies | parent | .69** | .13* | .56** | .40** |  |
| maladaptive strategies | child | .41** | .11 | .57** | .44** |  |
| adaptive strategies | parent | -.67** | -.15* | -.50** | -.37** |  |
| adaptive strategies | child | -.32** | -.15* | -.44** | -.30** |  |
| correlations with measures of externalizing and internalizing symptoms^b^ | | | | | | |
| ODD symptoms | parent | .84** | .32** | .67** | .52** |  |
| ODD symptoms | child | .58** | .12* | .73** | .59** |  |
| CD symptoms | parent | .56** | .17** | .42** | .31** |  |
| CD symptoms | child | .41** | .10 | .52** | .44** |  |
| ADHD symptoms | parent | .65** | .36** | .53** | .37** |  |
| ADHD symptoms | child | .47** | .24** | .62** | .52** |  |
| externalizing total symptoms | clinician | .78** | .42** | .68** | .54** |  |
| internalizing total symptoms | clinician | .62** | .21** | .48** | .32** |  |
| correlations with measures of health-related quality of life^c^ | | | | | | |
| total | parent | -.65** | -.35** | -.53** | -.43** |  |
| total | child | -.43** | -.20** | -.58** | -.46** |  |

*Note.* DADYS = Diagnostic Tool for Affective Dysregulation in Children. PQ = parent questionnaire. CQ = child questionnaire. CBCL = Child Behavior Checklist. ODD = oppositional defiant disorder. CD = conduct disorder. ADHD = attention-deficit/hyperactivity disorder.

^a^ assessed with the Questionnaire for the Regulation of Frustration in children (FRUST; Görtz-Dorten & Döpfner, 2021)

^b^ assessed with the Diagnostic System for Mental Disorders in children and adolescents according to ICD-10 and DSM-5 (DISYPS-III; Döpfner & Görtz-Dorten, 2017)

^c^ assessed with the KIDSCREEN (The KIDSCREEN Group Europe, 2006)

**p* < .05. ***p* < .01.
